# Antiretroviral Drug Use in a Cohort of HIV-Uninfected Women in the United States: HIV Prevention Trials Network 064

**DOI:** 10.1371/journal.pone.0140074

**Published:** 2015-10-07

**Authors:** Iris Chen, William Clarke, San-San Ou, Mark A. Marzinke, Autumn Breaud, Lynda M. Emel, Jing Wang, James P. Hughes, Paul Richardson, Danielle F. Haley, Jonathan Lucas, Anne Rompalo, Jessica E. Justman, Sally L. Hodder, Susan H. Eshleman

**Affiliations:** 1 Department of Pathology, Johns Hopkins University School of Medicine, Baltimore, Maryland, United States of America; 2 Vaccine and Infectious Disease Division, Fred Hutchinson Cancer Research Center, Seattle, Washington, United States of America; 3 Department of Biostatistics, University of Washington, Seattle, Washington, United States of America; 4 Department of Behavioral Sciences and Health Education, Rollins School of Public Health, Emory University, Atlanta, Georgia, United States of America; 5 Science Facilitation Department, FHI 360, Durham, North Carolina, United States of America; 6 Department of Medicine, Johns Hopkins University School of Medicine, Baltimore, Maryland, United States of America; 7 ICAP at Columbia University, Mailman School of Public Health, Columbia University, New York, New York, United States of America; 8 Clinical and Translational Science Institute, West Virginia University, Morgantown, West Virginia, United States of America; University of Pittsburgh, UNITED STATES

## Abstract

Antiretroviral (ARV) drug use was analyzed in HIV-uninfected women in an observational cohort study conducted in 10 urban and periurban communities in the United States with high rates of poverty and HIV infection. Plasma samples collected in 2009–2010 were tested for the presence of 16 ARV drugs. ARV drugs were detected in samples from 39 (2%) of 1,806 participants: 27/181 (15%) in Baltimore, MD and 12/179 (7%) in Bronx, NY. The ARV drugs detected included different combinations of non-nucleoside reverse transcriptase inhibitors and protease inhibitors (1–4 drugs/sample). These data were analyzed in the context of self-reported data on ARV drug use. None of the 39 women who had ARV drugs detected reported ARV drug use at any study visit. Further research is needed to evaluate ARV drug use by HIV-uninfected individuals.

## Introduction

The prevalence of antiretroviral (ARV) drug use among HIV-uninfected individuals is unclear. In the United States (US), ARV drugs are used by HIV-uninfected individuals for pre- and post-exposure prophylaxis (PrEP and PEP) [1–3]. ARV drugs, such as nucleoside/nucleotide reverse transcriptase inhibitors (NRTIs), are also used to treat chronic hepatitis B virus infection [4]. Several reports also indicate that some ARV drugs are used for recreational purposes [5,6]. ARV drug resistance can emerge if individuals become HIV infected while using ARV drugs.

HIV Prevention Trials Network (HPTN) 064 was an observational study conducted in 2009–2010 that assessed behaviors and HIV incidence among women in the US at increased risk for HIV infection who resided in areas of poverty and high HIV prevalence [7,8]. Women in HPTN 064 reported high prevalence of risky sexual behaviors and drug and alcohol use [7]. HIV incidence was estimated using a multifaceted approach that included analysis of HIV seroconversion [8]. The annual HIV incidence in the cohort was 0.32% (95% confidence interval [CI]: 0.14%-0.74%). One woman who acquired HIV infection during the study had undetectable HIV RNA at her first HIV positive visit and had emtricitabine (FTC) and tenofovir (TFV) detected in her plasma (unpublished data). These drugs are components of Truvada® (co-formulated FTC/TFV), which was later approved by the US Food and Drug Administration (FDA) for use as PrEP [1]. Some HIV-uninfected women in HPTN 064 also reported prior ARV drug use for PEP. In this report, we used a low cost, high-throughput ARV drug assay to estimate the prevalence and patterns of ARV drugs detected in plasma samples from HIV-uninfected women enrolled in HPTN 064.

## Methods

### Study cohort

HPTN 064 enrolled 2,099 women who reported no prior HIV diagnosis at ten urban and periurban communities in the US: Atlanta and Decatur, GA; Baltimore, MD; Durham and Raleigh, NC; Bronx and Harlem, NY; north and south Newark, NJ; and Washington, DC [7–9]. Women were recruited at community venues using time-space sampling and were followed for up to one year [10]. Study participants reported unprotected sex with a man and at least one other risk factor (individual or partner) for HIV infection in the six months prior to study enrollment. Participants completed audio computer-assisted self-interviews at each study visit (enrollment, 6 months, and 12 months). Women were asked about prior ARV drug use at the time of study enrollment (“Has the participant ever taken any antiretroviral medications?”) and at each follow-up visit (“Since her last visit, has the participant started taking any antiretroviral medications or changed antiretroviral medications?”). Participants who answered “Yes” were asked to specify the reasons for their ARV drug use (“Why did the participant take antiretroviral medications?”), which included taking ARV drugs for PrEP, PEP, treatment of HIV disease (i.e., ARV treatment [ART]; follow-up visits only), or “other” reasons. Women also received HIV testing and provided plasma samples for laboratory analyses at each study visit. This analysis included the 1,806 HIV-uninfected women who had a sample available from their last study visit. Randomly-selected enrollment samples from 369 of the 1,806 women (approximately 40 from each study site) were also tested to analyze ARV drug use at the time of study enrollment.

### Laboratory methods

Plasma samples were analyzed retrospectively for the presence of 16 ARV drugs: five NRTIs (FTC, TFV, lamivudine [3TC], stavudine, and zidovudine [ZDV]), two non-nucleoside reverse transcriptase inhibitors (NNRTIs; efavirenz [EFV] and nevirapine), and nine protease inhibitors (PIs; atazanavir, amprenavir, darunavir, lopinavir, indinavir [IDV], nelfinavir [NFV], saquinavir [SQV], tipranavir [TPV], and ritonavir [RTV]). Samples were prepared by combining 100 μL plasma with 300 μL acetonitrile containing 125 ng/mL deuterated morphine-d3. The resulting supernatant was then dried and reconstituted with 150 μL water. ARV drugs were detected using high-performance liquid chromatography (HPLC) coupled with high-resolution accurate mass (HRAM) mass spectrometry (MS; Exactive-Orbitrap; Thermo Scientific, Pittsburgh, PA). The mobile phase system consisted of 10 mM ammonium acetate and 0.1% ammonium hydroxide in methanol. Samples were introduced onto a 1.9-μm Hypersil Gold perfluorinated phenyl column at 100% aqueous composition; elution occurred during a 3.25 minute step-and-hold isocratic step to 100% methanol. Positive full-ion MS and all-ion fragmentation were conducted at 100,000 and 25,000 resolution, respectively; negative full-ion MS was conducted at 100,000 resolution. ZDV and EFV were detected using parent-ion scanning in negative ion mode and fragment identification in positive ion mode. All other ARV drugs were analyzed in positive ion mode for both the parent and fragment ions. The limit of identification for all ARV drugs in this assay was 10 ng/mL.

### Statistical methods

Associations between ARV drug detection and individual and partner characteristics were examined using Fisher’s exact and chi-square tests for categorical variables and Wilcoxon rank sum tests for continuous variables (SAS, version 9.2; SAS Institute, Cary, NC).

### Ethical considerations

All study participants provided written informed consent for participation in the HPTN 064 study. A Certificate of Confidentiality was obtained for HPTN 064, and the study was approved by institutional review boards at each study site and participating institution: Bronx-Lebanon Hospital Center; Columbia University Medical Center; Emory University; George Washington University; Johns Hopkins Medicine; New Jersey Medical School; and University of North Carolina at Chapel Hill.

## Results

### Study cohort

The HPTN 064 study is described in detail in a previous report [7]. In this study, 2,065 women were HIV uninfected at study enrollment [7,8]. Four women acquired HIV infection during study follow-up and were not included in this report; the remaining 2,061 women were HIV uninfected at the last study visit. Samples from 255 (12%) of the 2,061 women at the last study visit were not available for ARV drug testing (the study participants did not consent to additional testing or did not have plasma available). We analyzed the remaining 1,806 samples using a multi-drug assay, including 109 (6%) from the enrollment visit, 384 (21%) from the 6-month visit, and 1,313 (73%) from the 12-month visit. For comparison, 369 additional enrollment samples were tested from a random subset of participants.

### ARV drug detection

ARV drugs were detected in 39 (2%) of the 1,806 samples, including samples from 27 (15%) of the 181 women from Baltimore, MD and 12 (7%) of the 179 women from Bronx, NY (Table 1). ARV drugs were not detected in samples from women at the other eight communities. None of the 39 women who had ARV drugs detected reported ARV drug use at any study visit. Among the 1,767 women who did not have ARV drugs detected, nine (5%) reported ARV drug use at one of their study visits. These women were enrolled at five study sites (Decatur, GA; Raleigh, NC; Bronx and Harlem, NY; and Washington, DC). All nine women reported using ARV drugs for PEP; one woman also reported “intent to sell” when asked about other reasons for ARV drug use.

**Table 1:** Antiretroviral Drugs Detected in Plasma Samples Collected at the Last Study Visit.

	Overall (n = 1,806)	Baltimore (n = 181)	Bronx (n = 179)
ARV drug(s) detected	39 (2%)	27 (15%)	12 (7%)
EFV alone	10	5	5
NFV alone	9	9	-
IDV alone	5	1	4
SQV alone	5	5	-
EFV + IDV	3	-	3
TPV alone	2	2	-
NFV + SQV	2	2	-
EFV + NFV	1	1	-
ATV + IDV + NFV	1	1	-
EFV + IDV + NFV + SQV	1	1	-

The table shows the patterns of antiretroviral (ARV) drugs detected in plasma samples from HIV-uninfected participants enrolled in the HPTN 064 study. Samples were screened for the presence of five nucleoside/nucleotide reverse transcriptase inhibitors, two non-nucleoside reverse transcriptase inhibitors, and nine protease inhibitors. ARV drugs were detected in samples from participants enrolled at two of ten study communities, Baltimore, MD and Bronx, NY. Abbreviations: ARV, antiretroviral; EFV, efavirenz; NFV, nelfinavir; IDV, indinavir; SQV, saquinavir; TPV, tipranavir; ATV, atazanavir.

In Baltimore, 22 (81%) of the 27 women who had ARV drugs detected had a single drug detected (EFV or a PI alone); the PIs that were detected alone most frequently were NFV and SQV. The five remaining women (19%) had multiple ARV drugs detected (multiple PIs or EFV with one or more PIs). In Bronx, nine (75%) of the 12 women who had ARV drugs detected had a single drug detected (EFV or IDV). The remaining three women (25%) had EFV with IDV detected.

Univariate analyses were performed to evaluate the association of demographic and behavioral factors with ARV drug detection (Tables 2–4). In Baltimore, ARV drug detection was associated with race (P = 0.040), childhood abuse (odds ratio [OR]: -6.3; P = 0.049), and having a partner who was incarcerated within the past five years (OR: 4.7; 95% CI: 1.3–25.3; P = 0.008). In Bronx, ARV drug detection was associated with older age (mean age difference: 5.3 years; 95% CI: 1.0–9.6 years; P = 0.012). When results from both study sites were combined, ARV drug detection was significantly associated with childhood abuse (OR: 2.3; 95% CI: 1.1–5.0; P = 0.015).

**Table 2:** Association of Antiretroviral Drug Detection and Demographic Characteristics of HIV-uninfected Study Participants in Baltimore, MD and Bronx, NY.

		Baltimore			Bronx			Baltimore and Bronx		
		ARV drugs detected			ARV drugs detected			ARV drugs detected		
Baseline		Yes	No		Yes	No		Yes	No	
Characteristic		27	154	P value	12	167	P value	39	321	P value
Median age (IQR)		31 (25, 37)	35 (26, 41)	0.41	34 (27, 38)	26 (22, 32)	**0.011**	33 (26, 37)	29 (23, 39)	0.10
Race	Black	23 (85%)	143 (93%)	**0.040**	9 (75%)	106 (64%)	0.42	32 (82%)	249 (78%)	0.30
	White	2 (7%)	9 (6%)		1 (8%)	42 (25%)		3 (8%)	51 (16%)	
	Mixed	0 (0%)	2 (1%)		0 (0%)	3 (2%)		0 (0%)	5 (2%)	
	Other	2 (7%)	0 (0%)		2 (17%)	16 (10%)		4 (10%)	16 (5%)	
Hispanic ethnicity		1 (4%)	3 (2%)	0.48	6 (50%)	91 (55%)	0.76	7 (18%)	94 (29%)	0.14
Education	< HS grad	13 (48%)	76 (49%)	0.99	7 (58%)	70 (42%)	0.48	20 (51%)	146 (46%)	0.67
	HS grad	9 (33%)	51 (33%)		3 (25%)	47 (28%)		12 (31%)	98 (31%)	
	> HS grad	5 (19%)	27 (18%)		2 (17%)	50 (30%)		7 (18%)	77 (24%)	
Marital status	Single, sep, widowed, div	15 (56%)	98 (64%)	0.36	6 (55%)	88 (55%)	0.50	21 (55%)	186 (59%)	0.31
	Married or cohabitating	9 (33%)	49 (32%)		3 (27%)	58 (37%)		12 (32%)	107 (34%)	
	Other	3 (11%)	7 (5%)		2 (18%)	13 (8%)		5 (13%)	20 (6%)	
	Unknown	0	0		1	8		1	8	
Income	≤$10,000	5 (19%)	12 (8%)	0.12	1 (8%)	24 (14%)	0.61	6 (15%)	36 (11%)	0.84
	$10,001-$20,000	2 (7%)	12 (8%)		1 (8%)	18 (11%)		3 (8%)	30 (9%)	
	>$20,000	12 (44%)	54 (35%)		3 (25%)	64 (38%)		15 (39%)	118 (37%)	
	Unknown	8 (30%)	76 (49%)		7 (58%)	61 (37%)		15 (39%)	137 (43%)	
Food	Yes	14 (52%)	75 (49%)	0.76	9 (75%)	82 (49%)	0.08	23 (59%)	157 (49%)	0.24
insecurity	No	13 (48%)	79 (51%)		3 (25%)	85 (51%)		16 (41%)	164 (51%)	

The table shows the demographic characteristics of HIV-uninfected participants from Baltimore, MD and Bronx, NY whose plasma samples were screened for antiretroviral (ARV) drugs. Fisher’s exact, chi-square and Wilcoxon rank sum tests were used to analyze the association between these characteristics and ARV drug detection. P values <0.05 are bolded. Baseline characteristics are defined as follows: HS grad: high school graduation; sep: separated; div: divorced; cohabitating: not married but living with partner; income: annual household income; food insecurity: concerned about having sufficient food for self and family. Abbreviations: ARV: antiretroviral; IQR: interquartile range.

**Table 3:** Association of Antiretroviral Drug Detection with Behavioral Characteristics of HIV-uninfected Study Participants in Baltimore, MD and Bronx, NY.

	Baltimore			Bronx			Baltimore and Bronx		
	ARV drugs detected			ARV drugs detected			ARV drugs detected		
Baseline	Yes	No		Yes	No		Yes	No	
Characteristic	27	154	P value	12	167	P value	39	321	P value
Median number of partners (IQR)	2.0 (1.0, 6.0)	2.0 (1.0, 4.0)	0.74	2.5 (1.0, 3.0)	2.0 (1.0, 4.0)	0.99	2.0 (1.0, 3.0)	2.0 (1.0, 4.0)	0.63
Exchange sex for commodities^a^	13 (50%)	76 (51%)	0.92	3 (25%)	49 (29%)	1.00	16 (42%)	125 (40%)	0.76
Unknown HIV status of last partner	8 (30%)	66 (43%)	0.20	4 (33%)	56 (34%)	1.00	12 (31%)	122 (38%)	0.38
Condom use (vaginal)	4 (15%)	11 (7%)	0.25	4 (33%)	26 (16%)	0.12	8 (21%)	37 (12%)	0.12
Anal sex	12 (44%)	68 (44%)	0.98	5 (42%)	75 (45%)	0.83	17 (44%)	143 (45%)	0.91
Condom use (anal)^b^	0 (0%)	11 (16%)	0.20	0 (0%)	11 (15%)	1.00	0 (0%)	22 (15%)	0.13
Concurrency^a^	15 (56%)	72 (47%)	0.42	5 (42%)	68 (41%)	1.00	20 (51%)	140 (44%)	0.37
Self-reported STI^a^	4 (15%)	25 (16%)	1.00	1 (9%)	14 (8%)	1.00	5 (13%)	39 (12%)	0.80
Substance use	14 (52%)	86 (56%)	0.70	4 (33%)	69 (41%)	0.76	18 (46%)	155 (48%)	0.80
Binge drinking^a^	9 (33%)	46 (30%)	0.73	3 (25%)	56 (34%)	0.75	12 (31%)	102 (32%)	0.89
Drug use	11 (41%)	56 (36%)	0.66	1 (8%)	24 (14%)	1.00	12 (31%)	80 (25%)	0.43
Depressive symptoms^a^	16 (59%)	68 (45%)	0.18	3 (30%)	55 (35%)	1.00	19 (51%)	123 (40%)	0.19
Any history of abuse	11 (41%)	53 (34%)	0.53	6 (50%)	54 (32%)	0.22	17 (44%)	107 (33%)	0.20
Any childhood abuse	18 (67%)	71 (46%)	**0.049**	7 (58%)	69 (41%)	0.25	25 (64%)	140 (44%)	**0.015**

The table shows characteristics of HIV-uninfected participants who were enrolled in Baltimore, MD and Bronx, NY (limited to participants whose samples were screened for ARV drugs in this study). Study participants were asked to self-report characteristics within six months of enrollment, unless otherwise specified. Fisher’s exact, chi-square, and Wilcoxon rank sum tests were used to analyze the association between these characteristics and ARV drug detection. P values <0.05 are bolded. Baseline characteristics are defined as follows: Unknown HIV status of last partner: unknown HIV status of man with whom had last vaginal sex; Condom use (vaginal): Condom used with last vaginal sex; Condom use (anal): Condom used with last anal sex; Concurrency: self-report of sex with a man while involved in a sexual relationship with another man during the same period; Self-reported STI: Self-reported sexually-transmitted infection, including gonorrhea, syphilis, or chlamydia infection; Substance use: At least weekly substance use (including drug use or binge-drinking [≥4 drinks on 1 occasion]); Binge drinking: At least weekly binge-drinking (≥4 drinks on 1 occasion); Drug use: At least weekly drug use (excluding cannabis); Depressive symptoms: score ≥7 using the Center for Epidemiologic Studies Depression (CES-D) scale. Abbreviations: ARV: antiretroviral; IQR: interquartile range.

^a^Some participants did not respond to all of the questions asked. In these cases, the percentage was calculated among all of the respondents.

^b^This percentage was calculated among participants who reported ever having anal sex.

**Table 4:** Association of Antiretroviral Drug Detection with Behavioral Characteristics of Partners of HIV-uninfected Study Participants in Baltimore, MD and Bronx, NY.

	Baltimore			Bronx			Baltimore and Bronx		
	ARV drugs detected			ARV drugs detected			ARV drugs detected		
Baseline	Yes	No		Yes	No		Yes	No	
Characteristic	27	154	P value	12	167	P value	39	321	P value
HIV positive diagnosis	1 (4%)	1 (1%)	0.28	0 (0%)	2 (1%)	1.00	1 (3%)	3 (1%)	0.37
Reported STI	7 (26%)	18 (12%)	0.07	1 (8%)	16 (10%)	1.00	8 (21%)	34 (11%)	0.11
Illicit drug use	14 (52%)	75 (49%)	0.76	3 (25%)	46 (28%)	1.00	17 (44%)	121 (38%)	0.47
Binge-drinking	15 (56%)	81 (53%)	0.78	9 (75%)	115 (69%)	0.76	24 (62%)	196 (61%)	0.95
Alcohol dependence	16 (59%)	77 (50%)	0.37	6 (50%)	79 (47%)	0.86	22 (56%)	156 (49%)	0.34
Incarceration	24 (89%)	97 (63%)	**0.008**	7 (58%)	111 (67%)	0.55	31 (80%)	208 (65%)	0.07

The table shows characteristics of partners of HIV-uninfected participants who were enrolled in Baltimore, MD and Bronx, NY (limited to participants whose samples were screened for ARV drugs in this study). Study participants were asked to self-report characteristics of partners within six months of enrollment, unless otherwise specified. Fisher’s exact, chi-square, and Wilcoxon rank sum tests were used to analyze the association between these characteristics and ARV drug detection. P values <0.05 are bolded. Baseline characteristics are defined as follows: Reported STI: Reported partner sexually-transmitted infection, including gonorrhea, syphilis, or chlamydia infection; Substance use: At least weekly substance use (including drug use or binge-drinking [≥4 drinks on 1 occasion]); Binge drinking: ≥5 drinks on 1 occasion; Alcohol dependence: Cut Down, Annoyed, Guilty, and Eye Opener (CAGE) score ≥2; Incarceration: incarcerated during the past 5 years. Abbreviations: ARV: antiretroviral.

We also analyzed enrollment samples from a random subset of the HIV-uninfected women to assess ARV drug use prior to enrollment (369 women). This randomly-selected subset of women included 10 of the 41 women from Baltimore who had ARV drugs detected at the last study visit, and five of the 44 women from Bronx who had ARV drugs detected at the last study visit. ARV drugs were not detected at enrollment in any of 369 women. Only one of the 369 women reported any prior use of ARV drugs at her enrollment visit, indicating that she had used ARV drugs for PEP. Among the remaining 1,437 women in the cohort (those who did not have enrollment samples tested for ARV drugs), only three reported prior ARV drug use at the enrollment visit; in all three cases, the women indicated that they had used ARV drugs for PEP.

## Discussion

We found regionally-distinct patterns of ARV drug use among HIV-uninfected women in this observational cohort. Overall, 39 (2%) of the women had ARV drugs detected; all 39 women were from Baltimore or Bronx. Only EFV and PIs were detected. EFV was detected in both Baltimore and Bronx. In contrast, several PIs were detected in Baltimore, while only IDV was detected in Bronx.

Few studies have evaluated ARV drug use in HIV-uninfected women. At the time HPTN 064 was conducted (2009–2010), ARV drugs were not approved by the US FDA for PrEP; Truvada® (co-formulated FTC/TFV) was approved for PrEP in 2012 [1]. In contrast, EFV- and PI-based regimens with dual NRTI backbones were recommended for PEP by the US Department of Health and Human Services (DHHS) at that time [2,11]. The ARV drugs detected in this study, with the exception of TPV, were consistent with drugs that were recommended as part of PEP regimens during the study period. All of the HIV-uninfected women in HPTN 064 who reported any prior ARV drug use also indicated that the drugs had been used for PEP. We did not detect any NRTIs that are recommended components of triple-drug PEP regimens. It is possible that some women were taking NRTIs that were not detected because of the relatively short half-lives of these drugs. We also cannot exclude the possibility that plasma storage impacted ARV drug detection. However, the samples tested in this study were stored at -80°C prior to ARV drug testing, and several studies have documented the stability of ARV drugs in frozen plasma samples [12–15].

Individuals who become HIV infected while using ARV drugs are at risk for acquiring ARV drug resistance, and some ARV drugs are associated with toxic or unfavorable side effects and negative drug-drug interactions [16]. It is notable that most of the PIs detected in this study were not recommended for ART during the study period (e.g., due to toxicities and/or low efficacy) [17]. Several women in this study also had unusual combinations of ARV drugs detected (e.g., multiple PIs or EFV with one or more PIs). The US FDA and DHHS guidelines for PrEP, PEP, or ART do not recommend using more than one PI, unless a PI is being boosted by RTV, or using an NNRTI with a PI [1–3,11,16].

HIV-uninfected individuals may use ARV drugs for purposes other than HIV prevention. The NRTIs, 3TC and TFV, are recommended for treating chronic hepatitis B virus infection [4]; these NRTIs were not detected in this study. ARV drugs may also be used for recreational reasons. Reports indicate that EFV is sometimes used for its psychoactive effects [5], while PIs, most notably RTV, may be used to prolong the effects of certain recreational drugs [6]. In this study, we did not find any associations between ARV drug detection and self-reported individual or partner substance use.

Limited data are available about the prevalence of ARV drug use among HIV-uninfected individuals. Recent studies report that PrEP and PEP use has increased over time [18,19]. However, these studies are limited to electronic prescription data, which may not reflect actual PrEP and PEP use in the community. HIV-uninfected individuals may acquire ARV drugs from health care providers or from other sources. ARV drug sharing for PrEP and PEP between HIV-infected and HIV-uninfected men who have sex with men has been documented in the US [20,21]. Some reports also indicate that ARV drugs may be traded, sold, or purchased in illicit marketplaces [22,23]. Notably, one woman in HPTN 064 not only reported acquiring ARV drugs for PEP, but also reported an intention to sell the drugs when asked about other reasons for her ARV drug use.

In this study, we used a low cost, high-throughput assay to screen for multiple ARV drugs in more than 2,000 samples. This assay makes it feasible to perform large-scale studies to gain information about the prevalence and patterns of ARV drug use in different populations. This assay also provides an objective, biomedical measure of ARV drug use. None of the women who had ARV drugs detected in this study reported any prior ARV drug use. Nondisclosure of ARV drug use has been reported in both clinical and research settings [24–27]. Women in HPTN 064 who had ARV drugs detected may not have been known that these were ARV medications or may not have been familiar with the term “antiretroviral”, which was used in the study questionnaire. As PrEP and PEP use continues to expand, further research is needed to explore the extent of ARV drug use among HIV-uninfected women, the means by which those drugs are acquired, the reasons for their use, and their impact on HIV drug resistance.

## Abbreviations

3TC: lamivudine
ARV: antiretroviral
DHHS: Department of Health and Human Services
EFV: efavirenz
FDA: Food and Drug Administration
FTC: emtricitabine
HPTN: HIV Prevention Trials Network
IDV: indinavir
NFV: nelfinavir
NNRTI: non-nucleoside reverse transcriptase inhibitor
NRTI: nucleoside/nucleotide reverse transcriptase inhibitor
PEP: post-exposure prophylaxis
PrEP: pre-exposure prophylaxis
PI: protease inhibitor
RTV: ritonavir
SQV: saquinavir
TFV: tenofovir
TPV: tipranavir
US: United States
ZDV: zidovudine
